# Development of trofinetide for the treatment of Rett syndrome: from bench to bedside

**DOI:** 10.3389/fphar.2023.1341746

**Published:** 2024-01-22

**Authors:** Melissa Kennedy, Larry Glass, Daniel G. Glaze, Steve Kaminsky, Alan K. Percy, Jeffrey L. Neul, Nancy E. Jones, Daniela Tropea, Joseph P. Horrigan, Paige Nues, Kathie M. Bishop, James M. Youakim

**Affiliations:** ^1^ International Rett Syndrome Foundation, Cincinnati, OH, United States; ^2^ Neuren Pharmaceuticals Ltd., Melbourne, VIC, Australia; ^3^ Texas Children’s Hospital, Baylor College of Medicine, Houston, TX, United States; ^4^ Division of Pediatric Neurology, University of Alabama at Birmingham, Birmingham, AL, United States; ^5^ Vanderbilt Kennedy Center, Vanderbilt University Medical Center, Nashville, TN, United States; ^6^ Institute of Neuroscience, Trinity College, Dublin, Ireland; ^7^ Department of Psychiatry and Behavioral Sciences, Duke University, Durham, NC, United States; ^8^ Acadia Pharmaceuticals Inc., San Diego, CA, United States

**Keywords:** trofinetide, Rett syndrome, Neuren Pharmaceuticals, Acadia Pharmaceuticals, International Rett syndrome Foundation, LAVENDER, LILAC, LILAC-2

## Abstract

Rett syndrome (RTT) is rare neurodevelopmental disorder caused by mutations in the *MECP2* gene that encodes methyl-CpG-binding protein 2 (MeCP2), a DNA-binding protein with roles in epigenetic regulation of gene expression. Functional loss of MeCP2 results in abnormal neuronal maturation and plasticity, characterized by loss of verbal communication and loss of fine and gross motor function, among others. Trofinetide, a synthetic analog of glycine-proline-glutamate, was approved by the US Food and Drug Administration for the treatment of RTT in adult and pediatric patients aged 2 years and older. Here, we present the development of trofinetide from bench research to clinical studies and emphasize how the collaboration between academia, the pharmaceutical industry, and patient advocacy led to the recent approval. The bench-to-bedside development of trofinetide underscores the value of collaboration between these groups in the development and approval of treatments for rare diseases.

## 1 Introduction

Rett syndrome (RTT) is a rare neurodevelopmental disorder that primarily affects females but also some males (Amir et al., 1999). Most cases of RTT are caused by *de novo* loss-of-function mutations in the *MECP2* gene that encodes methyl-CpG-binding protein 2 (MeCP2), a DNA-binding protein with roles in epigenetic regulation of gene expression. Functional loss of MeCP2 results in abnormal neuronal maturation and plasticity (Amir et al., 1999; Belichenko et al., 2009; Baj et al., 2014; Bedogni et al., 2016). RTT is characterized by a broad set of core symptoms including loss of verbal communication with limited nonverbal skills, loss of fine and gross motor function (including purposeful hand use), behavioral issues, seizures, hand stereotypies, and gastrointestinal problems (Neul et al., 2010; Motil et al., 2012; Fu et al., 2020a; Fu et al., 2020b).

Until recently, there has not been an approved treatment for the core symptoms of RTT and treatment has relied on a polypharmacy approach that seeks to manage individual symptoms (May et al., 2023). Trofinetide, a synthetic analog of glycine-proline-glutamate (GPE), was approved by the US Food and Drug Administration (FDA) in March 2023 for the treatment of RTT in adult and pediatric patients aged 2 years and older (Acadia Pharmaceuticals, 2023). Here, we present the bench-to-bedside development of trofinetide to highlight the years of scientific and clinical research that led to the recent FDA approval. We also highlight the academic research that led to the design of trofinetide, the contributions of Neuren Pharmaceuticals and Acadia Pharmaceuticals in the clinical development program and FDA approval of trofinetide, and the involvement of the International Rett Syndrome Foundation (IRSF) in advocacy of patients and families living with RTT.

## 2 Bench research: discovery of GPE, applicability in RTT, and development of trofinetide

Insulin-like growth factor-1 (IGF-1), a naturally occurring protein in the brain, has neuroprotective properties (Gluckman and Ambler, 1992). IGF-1 is cleaved to the neuroactive tripeptide GPE (Sara et al., 1989). GPE was found to partially rescue immature synaptic function and organization in a MeCP2 mutant RTT mouse model, suggesting it had applicability for the treatment of RTT. Additionally, GPE extended the life span of the mutant RTT mice, improved locomotor function, and ameliorated breathing patterns, among others (Tropea et al., 2009). GPE is not enzymatically stable and has a plasma half-life <5 min, requiring intravenous infusion for stable neuroprotection (Batchelor et al., 2003). Trofinetide was discovered as an analog of GPE that improved proteolytic stability and bioavailability of the molecule (Brimble et al., 2005; Harris et al., 2005; Lai et al., 2005; Trotter et al., 2005; Harris and Brimble, 2006; Bickerdike et al., 2009; Hung et al., 2009).

## 3 Trofinetide clinical program in RTT

Trofinetide was initially investigated in traumatic brain injury *in vitro* and *in vivo* by Neuren Pharmaceuticals. In these models, trofinetide was found to significantly attenuate apoptosis in primary striatal cultures and reduce the level of expression of genes associated with inflammation, necrosis, and apoptosis (Lu et al., 2009; Wei et al., 2009). These findings motivated Neuren Pharmaceuticals to study trofinetide in RTT.

Neuren Pharmaceuticals conducted two phase II clinical trials of trofinetide in individuals with RTT. RETT-001 was a double-blind, placebo-controlled, dose-escalation study of 56 adolescent and female adults with RTT that compared trofinetide (35 and 70 mg/kg twice daily) with placebo for 28 days (Glaze et al., 2017). Clinical benefit was observed for the trofinetide 70 mg/kg dose compared with placebo across multiple domains of impairment, including communication, behavior, breathing abnormalities, hand movements or function, motor or muscular dysfunction, and seizures. Trofinetide was well tolerated in this study. RETT-002 was a double-blind, placebo-controlled, parallel-group study of 82 girls and adolescents with RTT comparing trofinetide (50, 100, and 200 mg/kg twice daily) with placebo for 42 days (Glaze et al., 2019). Trofinetide 200 mg/kg twice daily demonstrated statistically significant efficacy compared with placebo (*p* < 0.05) on three RTT-specific assessments: the Rett Syndrome Behaviour Questionnaire (RSBQ), Clinical Global Impression-Improvement (CGI-I), and RTT-Clinician Domain Specific Concerns–Visual Analog Scale (RTT-DSC-VAS). Trofinetide was well tolerated in this study.

These phase II trials were instrumental in establishing a consistent clinical trial culture in RTT. The principal investigators were seasoned RTT clinicians and scientists who participated in preceding natural history studies of RTT that identified potential outcome measures that would be suitable for use in clinical trials. These outcome measures were refined for use in the phase II studies and included clinician-and caregiver-assessed endpoints that spanned syndrome-specific and general measures of efficacy. Additionally, endpoints were developed during the phase II trials that measured symptoms of RTT. An assessment of individual symptom domains of RTT (hand use, ambulation, seizures, autonomic features, behavior, attentiveness, social interaction, and language/communication) was developed using a visual analog scale (RTT-DSC-VAS). The CGI-I is a measure of global clinical improvement that has been widely used in clinical trials of central nervous system disorders and neurodevelopmental disorders (Neul et al., 2015; Neul et al., 2022). RTT-specific scoring anchors were developed to make the CGI-I rating scale relevant to RTT (Neul et al., 2015). Training and scoring calibration procedures were developed and used in the phase II studies to ensure consistency across the clinician raters. The experience with the assessment in the phase II studies and the established training procedures were important for its acceptability for the phase III program.

Acadia Pharmaceuticals acquired the rights to trofinetide in North America and executed the trofinetide phase III clinical trial program. Weight-based dosing in the phase III trials was intended to achieve the target exposure identified as potentially efficacious based on the phase II data. Trofinetide was given at 30 mL (6 g), 40 mL (8 g), 50 mL (10 g), or 60 mL (12 g) twice daily for participants weighing 12–20, >20–35, >35–50, and >50 kg, respectively (equivalent to a range of 200–500 mg/kg twice daily). The new approach to weight-based dosing increased consistency of dosing, simplified administration for caregivers, and reduced drug waste. The CGI-I and RSBQ were chosen as the coprimary efficacy endpoints in the phase III clinical program to capture clinician and caregiver perspectives of the efficacy of trofinetide in patients with RTT (Neul et al., 2022). The CGI-I was chosen for clinicians to measure the overall improvement of patients with the same RTT-specific scoring anchors used in the phase II study (Neul et al., 2015; Neul et al., 2022). The RSBQ, a validated caregiver assessment accepted by the FDA as an endpoint in RTT clinical trials, was chosen for caregivers to measure improvement in specific behaviors and symptom domains associated with RTT. The symptoms assessed are general mood, breathing problems, hand behaviors, repetitive face movements, body rocking and expressionless face, night-time behaviors, fear/anxiety, and walking/standing (Mount et al., 2002).

The Social composite score from the Communication and Symbolic Behavior Scales Developmental Profile™ Infant-Toddler Checklist (CSBS-DP-IT Social) was the key secondary endpoint of the phase III trials (Neul et al., 2022). This caregiver assessment was chosen as an endpoint to reflect that improvement in the ability to communicate is often the most important goal for caregivers in the treatment of RTT (Urbanowicz et al., 2016). The CSBS-DP-IT Social composite score includes the modalities most commonly used by people with RTT to communicate, such as emotion and eye gaze and gestures (Didden et al., 2010). Other efficacy endpoints were chosen with consideration of symptom prevalence in RTT, their functional impact, and the importance of the symptom as a treatment target for caregivers and clinicians (Neul et al., 2022). Four novel, standalone RTT clinician ratings were developed in place of the RTT-DSC-VAS and used as secondary endpoints–they focused on assessment of hand function (RTT-HF), ambulation and gross motor skills (RTT-AMB), communication (RTT-COMC), and verbal communication (RTT-VCOM) (Neul et al., 2022). The endpoints were chosen to have a low burden to the clinician, caregiver, and individuals with RTT.

The trofinetide clinical program executed by Acadia Pharmaceuticals consisted of several studies. LAVENDER was a phase III, randomized, double-blind, placebo-controlled study (Neul et al., 2023). The study evaluated the efficacy and safety of trofinetide, compared with placebo, in females with RTT aged 5–20 years for 12 weeks. In total, 184 females were enrolled in the study. LAVENDER met the RSBQ and CGI-I coprimary efficacy endpoints, pointing to benefit with trofinetide in treating the core symptoms of RTT. Additionally, trofinetide improved communication modalities compared with placebo in the participants of the study based on the CSBS-DP-IT Social composite score results. Overall, trofinetide was safe and well tolerated; diarrhea and vomiting were the most common treatment-emergent adverse events (TEAEs) with trofinetide (80.6% and 26.9%, respectively); most reports of diarrhea and vomiting were of mild or moderate severity (97.3% and 96.0%, respectively). Diarrhea management recommendations were developed to prevent and manage this adverse event (Marsh et al., 2023). LILAC was a phase III open-label extension study of LAVENDER that evaluated the safety and efficacy of trofinetide with up to 40 weeks of treatment (Percy et al., 2023). Overall, 154 females with RTT, aged 5–21 years, were enrolled in LILAC. In this study, open-label treatment with trofinetide continued to improve symptoms of RTT demonstrated on the RSBQ and the CGI-I. Safety and tolerability were consistent with the LAVENDER study. Further long-term evaluation of the safety and efficacy of trofinetide in RTT continued with LILAC-2, a phase III, open-label extension study of LILAC that extended treatment for ≤32 months (ClinicalTrials, 2023).

The youngest age allowed for enrollment in LAVENDER was 5 years, as participants were required to be ≥ 6 months post RTT regression. Inclusion of patients younger than 5 years of age in LAVENDER would have resulted in difficulty in evaluation of endpoints, as patients who are in the RTT regression period might show great variability in RTT symptoms. However, data supporting the use of trofinetide in this younger patient population was needed. DAFFODIL was a phase II, open-label study that evaluated the safety and efficacy in patients with RTT aged 2–4 years who were required to be post a regression period (Percy et al., 2022). The study enrolled 14 girls, treated with open-label trofinetide for approximately 2 years. DAFFODIL consisted of two treatment periods: period A, which lasted 12 weeks and was designed to evaluate dosing, safety, and exploratory efficacy endpoints, and period B, designed to assess long-term safety and efficacy for approximately 21 months. The results of period A were consistent with the findings of the LAVENDER study. Overall, trofinetide was safe and well tolerated; diarrhea and vomiting were the most common TEAEs reported with trofinetide (64.3% and 35.7%, respectively). All reports of diarrhea and vomiting were of mild or moderate severity. Trends toward improvements in efficacy were observed by week 12 of trofinetide treatment. Population pharmacokinetic analysis confirmed that trofinetide weight-banded dosing achieved the target exposure range in this young patient population.

Acadia Pharmaceuticals managed logistics to facilitate participation in all trials, such as transportation and accommodation for families during trial visits and shipment of trofinetide to their homes–these efforts ensured that participants remained engaged during the trial by minimizing the demands imposed on them. Acadia submitted a New Drug Application (NDA) to FDA in 2022, which led to the approval of trofinetide by the FDA on 10 March 2023 for the treatment of RTT in adults and pediatric patients 2 years of age and older (Acadia Pharmaceuticals, 2023).

## 4 Role of patient advocacy in the clinical development of trofinetide

IRSF has been a trusted advocate and voice for families of patients with RTT since 1983; their efforts in education of families, research, and advocacy contributed to the success of the trofinetide clinical program. Understanding the critical importance of a research network, IRSF partially funded the Rett Syndrome Natural History study, which became a pilot program that built relationships with investigators and trust with families. IRSF fostered relationships between clinicians who were treating RTT–often investigators of the Rett Syndrome Natural History Study–and Neuren Pharmaceuticals and Acadia Pharmaceuticals, leading to identification of sites for clinical studies. Furthermore, conducting the Rett Syndrome Natural History Study allowed for coordination and standardization of future clinical trial sites and further development of endpoints used in clinical trials. IRSF partially funded early clinical trials of trofinetide and facilitated participant recruitment in later clinical trials by providing education regarding clinical trial involvement to caregivers of individuals with RTT. Caregiver education included information on clinical trial design, emphasizing the rationale for inclusion/exclusion criteria that may not be readily understood by all families and the purpose of having a placebo arm in LAVENDER. IRSF was also in constant communication with families on the progress of the clinical trials, particularly during the COVID-19 pandemic. Furthermore, IRSF continues to be committed to raising the voices of patients with RTT, evidenced by their coordination of an externally led, patient-focused Drug Development Meeting in 2023. This meeting was attended by hundreds of caregivers of patients with RTT and allowed families to share their perspective on what is important to them as therapies for RTT are considered for approval.

## 5 Discussion

The success of the trofinetide clinical program involved the collaborative work of academia, the pharmaceutical industry, and patient advocacy. Academic research identified the role of IGF-1 pathway in RTT, which led to the identification of GPE as a potential treatment for RTT and the use of trofinetide to treat RTT. Neuren Pharmaceuticals conducted the phase II trials of trofinetide in RTT and Acadia Pharmaceuticals developed and conducted the trofinetide phase III clinical program, as well as the chemistry, manufacturing, and controls activities, and managed the regulatory strategy that culminated in the approval of trofinetide for the treatment of RTT. IRSF partially supported the Rett Syndrome Natural History Study, which set the stage for sites and endpoints of trofinetide clinical trials. IRSF also acted as a facilitator between academics, the pharmaceutical industry, and families of patients with RTT to foster effective collaboration between all parties. The approval of trofinetide in RTT highlights the importance of coordination between academia, the pharmaceutical industry, and patient advocacy for the development and approval of treatments for rare diseases.

## Data Availability

The original contributions presented in the study are included in the article/Supplementary material, further inquiries can be directed to the corresponding author.
